# Surgery for Chronic Postinfarct Ventricular Pseudoaneurysm Using 3-Dimensional Printing

**DOI:** 10.1016/j.atssr.2024.07.014

**Published:** 2024-07-31

**Authors:** Kevin Wang, Abel Cherian, Alex Ryan, Yash Rohilla, Chidiebere Peter Echieh, Michael D. Seckeler, Toshinobu Kazui

**Affiliations:** 1 Department of Surgery, Banner University Medical Center—Tucson, Tucson, Arizona; 2 College of Medicine—Tucson, University of Arizona, Tucson, Arizona; 3 College of Science, University of Arizona, Tucson, Arizona; 4 Department of Cardiothoracic Surgery, University of Arizona, Tucson, Arizona; 5 Department of Pediatrics (Cardiology), University of Arizona, Tucson, Arizona; 6 Division of Cardiothoracic Surgery, Department of Surgery, Banner University Medical Center—Tucson, Tucson, Arizona

## Abstract

The use of 3-dimensional printing allows for preoperative planning in complex cardiac surgery. It provides a digital and physical model of patient-specific anatomy that may aid in decision making. Our case describes a 64-year-old patient with a late presentation of myocardial infarction complicated by the development of chronic postinfarction ventricular septal defect with interventricular pseudoaneurysm. We utilized a 3-dimensional-printed model of the patient’s heart to identify the location of the defect preoperatively. From this case, we believe that 3-dimensional modeling may aid preoperative planning, particularly in complex anatomy. Time, cost, and precision are limitations to the routine clinical application of this technology.

Three-dimensional (3D) printing creates a digital and physical model of patient-specific anatomy and may inform preoperative surgical planning.^1^ We report a case of chronic post-infarction ventricular septal defect (VSD) with formation of interventricular pseudoaneurysm that required use of 3D printing to define anatomy. Three-dimensional printing identified, preoperatively, the orifice of interventricular pseudoaneurysm. Given the complexity of this patient’s anatomy, we found preoperative 3D printing helpful in informing our decision to successfully treat the patient using an extended sandwich patch technique.

We managed a 64-year-old male patient with progressive dyspnea and chest pain following a 2-month history of acute ST elevation myocardial infarction with occlusion of the right coronary artery which was stented. He subsequently developed postinfarction VSD. He presented to us in heart failure. Past medical history includes hypertension, hyperlipidemia, obstructive sleep apnea, and tobacco use. Physical exam demonstrated jugular venous pressure of 10 cm H_2_O with a 3/6 holosystolic murmur. Cardiac magnetic resonance imaging demonstrated a hemodynamically significant left-to-right shunt localized to the basal-to-mid inferoseptal wall. His Qp/Qs ratio was 1.64 on magnetic resonance imaging. Additionally, the right ventricle (RV) was severely dilated, with reduced ejection fraction of 40%. Cardiac computed tomography corroborated these findings, demonstrating an aneurysmal inferoseptal wall with a significant interventricular pseudoaneurysm (Figure 1). VSD size was 1.38 cm^2^ on the left ventricle (LV) and 0.3 cm^2^ on the RV.

**Figure 1:**
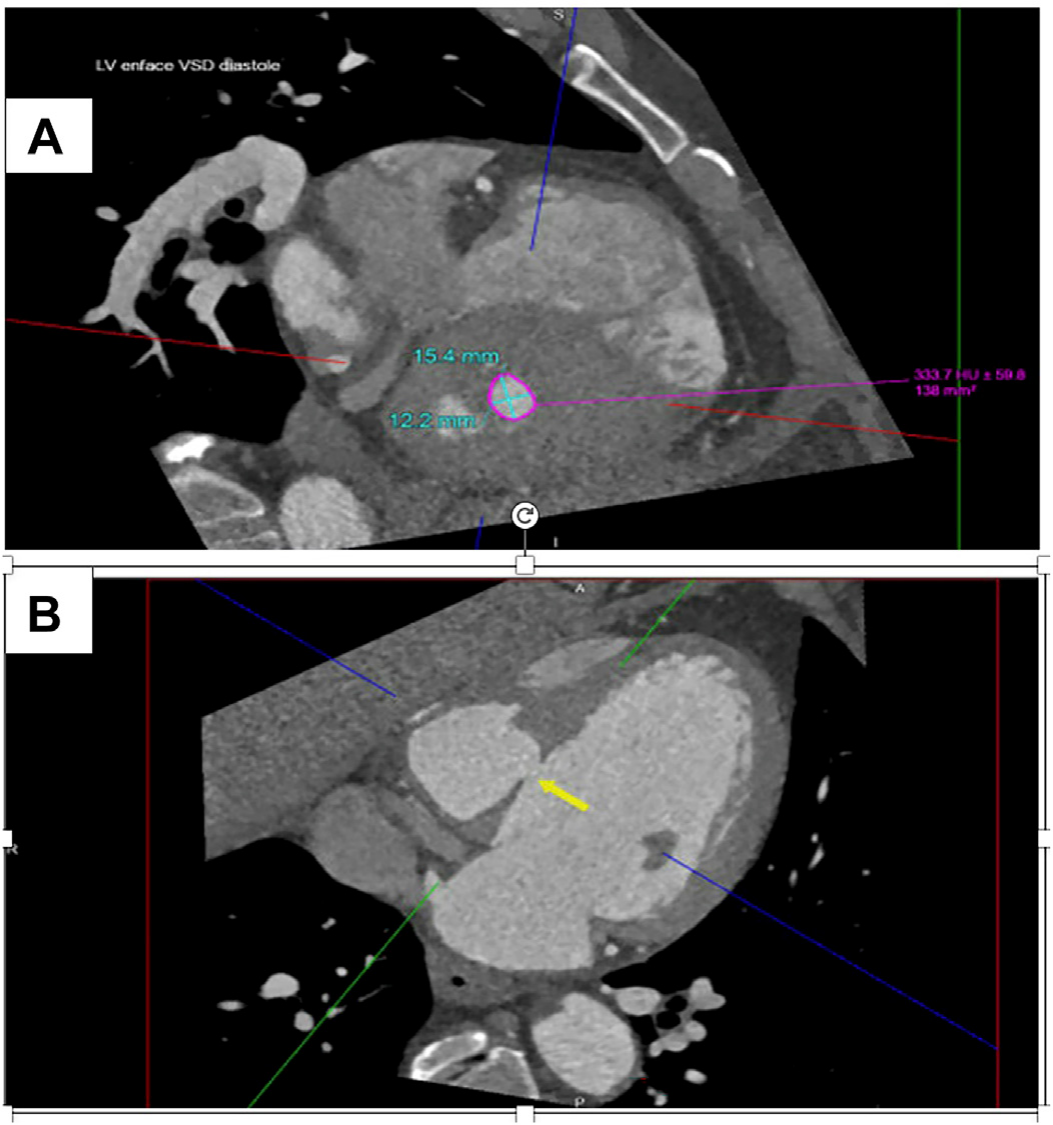
Computed tomography image showing postinfarction ventricular septal defect. (A) shows the Ventricular Septal Defect (VSD) as seen enface from the left ventricle (LV) and the dimensions of the VSD in diastole (blue X). (B) shows the location of the defect along the interventricular septum and shunt across the septum (yellow arrow).

As the pseudoaneurysm was large and astride the interventricular septum, it was critical to identify the orifices and surrounding structures in relation to the interventricular pseudoaneurysm. To facilitate the understanding of surrounding structure, we performed a virtual 3D reconstruction generated from the cardiac computed tomography using Vitrea v7.15 (Canon Medical Informatics). Stereolithography files were pre-processed in Autodesk Meshmixer v3.5 and 3D-printed using polylactic acid on a Dremel Idea Builder-3D40 (Figure 2).

**Figure 2:**
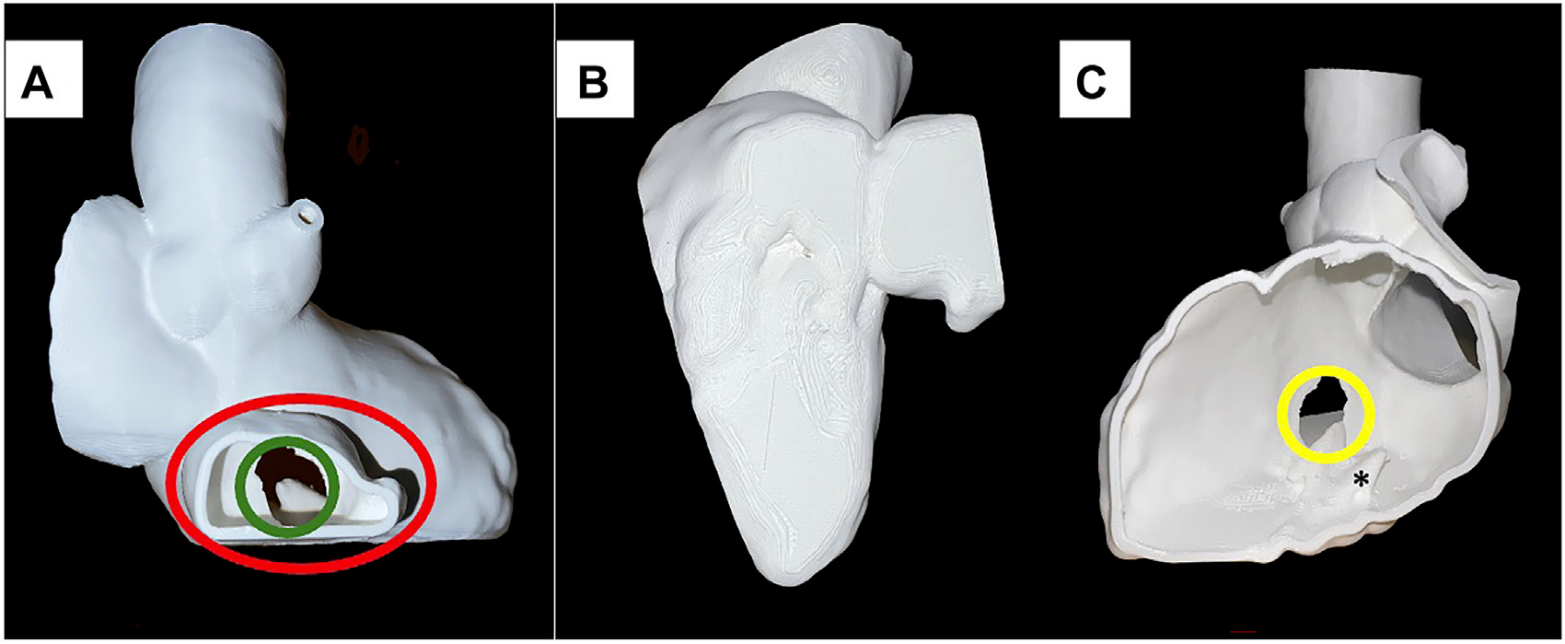
(A) Three-dimensionally printed model of right ventricle toward the ventricular septum showing the pseudoaneurysm (red) ventricular septal defect within it (green circle). (B) Posterolateral view of the heart demonstrating the pseudoaneurysm into the right ventricle (right ventricle walls cut away). (C) View from the left ventricle toward the ventricular septum (posterior wall cut away) showing the ventricular septal defect (yellow circle) in relation to the mitral valve papillary muscles (∗).

The 3D-printed model demonstrated the interventricular pseudoaneurysm in the RV. The entrance of the LV side was located 2 cm inferior to the mitral valve annulus and 2 cm medial from posteromedial papillary muscle. The interventricular pseudoaneurysm on the RV was located 2 cm below the tricuspid annulus. The exit of the interventricular pseudoaneurysm was close to the RV inferior wall. Based on this preoperative assessment, we decided to approach from the base of the RV inferior wall and close the postinfarction VSD with 2 large patches from the RV and LV side.

Through a right ventriculostomy made 1 cm medial to the posterior descending artery we visualized the VSD (Figures 3A, 3B) as identified preoperatively in the 3D-printing model. We excised the entrance of the aneurysm from the RV side to obtain access. The rest of the aneurysm wall was left for reinforcement of patch closure. The septum was cleared to allow the patch through the hole (Figure 3C), without extending the incision toward the base of LV. The dimensions of RV and LV defects were 0.9 x 0.5 cm and 1.5 x 1.0 cm, respectively. Using the aneurysm wall as reinforcement, an oval-shaped 5.0 x 6.0 cm bovine-pericardial patch was sutured on the LV side through the postinfarction VSD. The sutures for the base of the LV and anterior side were placed through the LV septum, avoiding the mitral valve annulus. All transseptal sutures from the LV were passed also through the corresponding margin of the RV patch and tied. A second patch was anchored by sutures through the free wall of the RV. Those sutures brought out of the free wall of the LV were secured through Teflon (DuPont) felt pledgets. After securely tying all transseptal sutures, the transmural sutures were placed from the RV through the remaining margin of the second patch and brought out of the heart. BioGlue (CryoLife, Inc) was used to reinforce the patch and minimize the residual shunt. Right ventriculotomy was closed with 3-0 Prolene (Ethicon) mattress and over-and-over sutures with felt strip reinforcement (Figure 3D). Postoperative course was uneventful; on 90-day follow-up, the patient reported improved dyspnea and magnetic resonance imaging demonstrated no residual shunting.

**Figure 3:**
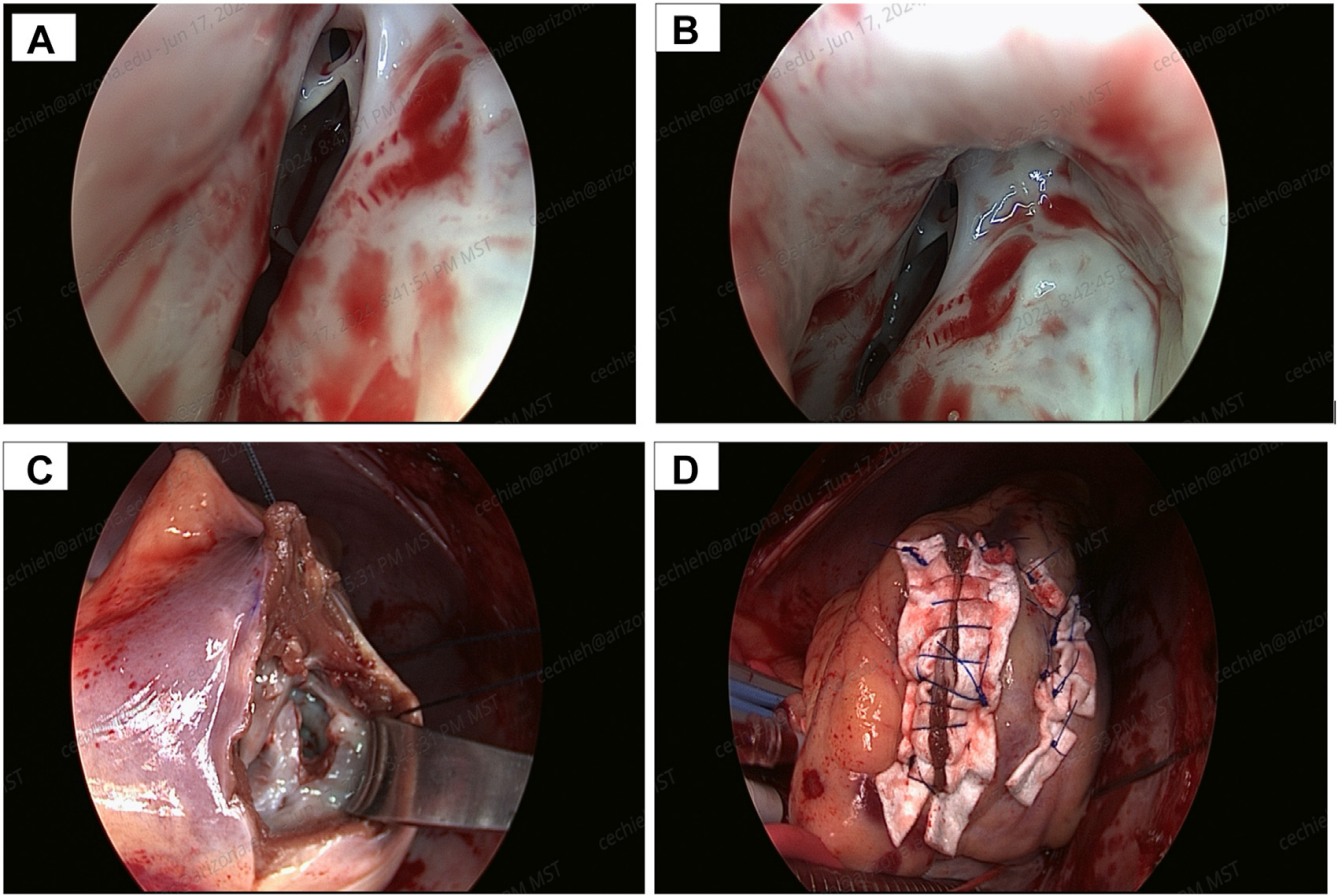
(A) Intraoperative picture showing the relationship of the ventricular septal defect with the papillary muscles. (B) Intraoperative picture showing the orifice of the pseudoaneurysm. (C) The ventricular septal defect before patch closure. (D) Reconstruction of the right ventriculostomy.

## Comment

Three-dimensional printing in cardiovascular interventions has been used for preoperative planning, medical teaching, and clinical consultations.^2^ Visualizing anatomy is critical to surgery, with 3D printing providing digital and physical preoperative anatomical adjunct. It allows for the creation of patient specific anatomic models prior to surgery. The process involves segmentation of the anatomy of interest, creation of stereolithography files, cleaning/preparation, and printing.^3^

This case report highlights the use of 3D printing in preoperative planning for a rare case of chronic postischemic VSD with development of an interventricular pseudoaneurysm. Use of 3D printing allowed preoperative definition of the location of the VSD in relation to the pseudoaneurysm and papillary muscles that would have been otherwise difficult to define. Specifically, the model portrayed the defect and the distance of the postinfarct VSD from mitral valve annulus and papillary muscles, thus removing any intraoperative difficulty identifying the defect. The 3D-printed model facilitated clear assessment of the VSD and the interconnections through the interventricular septum via a pseudoaneurysm. Preoperative identification of posterior location of the defect enabled surgical planning and adoption of inferior approach to the heart through the posterior aspect of the RV medial to the patent ductus arteriosus. With this preoperative assessment, we were able to optimize surgical exposure and place sutures through patches accurately. This case report documents the use of 3D printing for the preoperative planning of pseudoaneurysm repair.

We preferred to use the Dremel Idea Builder for this case because of its availability and our access to expertise in this technology.^4^^,^^5^ The median time to print with this technology is 3.83 hours (interquartile range, 3.35-7.02 hours), with a unit material cost of $2.84.^6^ A major limitation of the use of 3D printing in surgical planning for postinfarct complications is the time required for printing. Factors limiting the application of 3D printing to cardiac surgery include precision, preparation time, and cost.
